# Crystal structure of 8-eth­oxy-3-(4-nitro­phen­yl)-2*H*-chromen-2-one

**DOI:** 10.1107/S2056989015019325

**Published:** 2015-10-17

**Authors:** Shashikanth Walki, S. Naveen, S. Kenchanna, K. M. Mahadevan, M. N. Kumara, N. K. Lokanath

**Affiliations:** a Department of Chemistry, Kuvempu University, P. G. Centre, Kadur 577 548, India; b Institution of Excellence, University of Mysore, Manasagangotri, Mysore 570 006, India; c Department of Chemistry, Yuvaraja’s College, University of Mysore, Mysore 570 005, India; d Department of Studies in Physics, University of Mysore, Manasagangotri, Mysore 570 006, India

**Keywords:** crystal structure, coumarin, chromen, C—H⋯O hydrogen bonds, π–π stacking

## Abstract

In the title compound, C_17_H_13_NO_5_, the coumarin ring system is essentially planar (r.m.s. deviation = 0.008 Å). The nitro­phenyl ring makes a dihedral angle of 25.27 (9)° with the coumarin ring plane. The nitro group is almost coplanar with the phenyl ring to which it is attached, making a dihedral angle of 4.3 (3)°. The eth­oxy group is inclined to the coumarin ring plane by 4.1 (2)°. Electron delocalization was found at the short bridging C—C bond with a bond length of 1.354 (2) Å. In the crystal, mol­ecules are linked *via* C—H⋯O hydrogen bonds, forming sheets in the *bc* plane. The sheets are linked *via* π–π stacking [centroid–centroid distances = 3.5688 (13) and 3.7514 (13) Å], forming a three-dimensional structure.

## Related literature   

For coumarin derivatives as fluorescent brighteners, see: Tian *et al.* (2000 ▸). For details of natural or synthetic coumarins which inhibit lipid peroxidation and scavenge hydroxyl radicals and superoxide anions, see: Naveen *et al.* (2007 ▸). For further details of our research on coumarins, see: Naveen *et al.* (2006*a* ▸,*b* ▸).
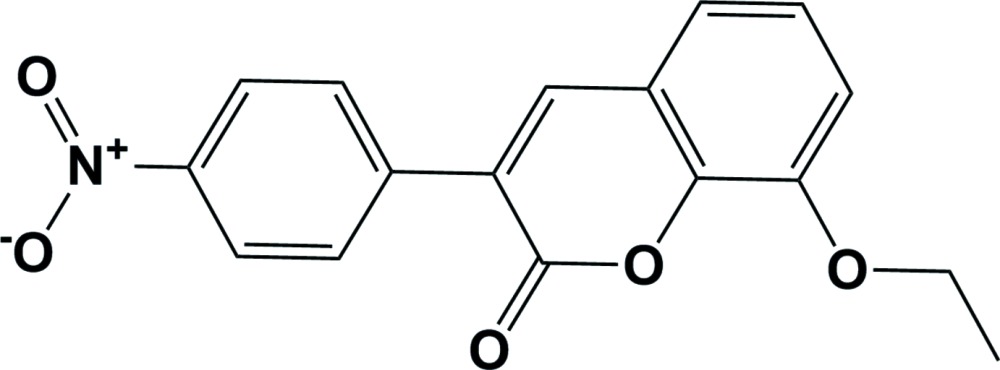


## Experimental   

### Crystal data  

C_17_H_13_NO_5_
*M* *_r_* = 311.28
Orthorhombic, 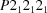

*a* = 6.8118 (9) Å
*b* = 13.6726 (18) Å
*c* = 15.909 (2) Å
*V* = 1481.7 (3) Å^3^
*Z* = 4
Cu *K*α radiation
μ = 0.87 mm^−1^
*T* = 296 K
0.29 × 0.26 × 0.21 mm

### Data collection  

Bruker X8 Proteum diffractometer
Absorption correction: multi-scan (*SADABS*; Bruker, 2013 ▸) *T*_min_ = 0.786, *T*_max_ = 0.838
6729 measured reflections
2371 independent reflections
2225 reflections with *I* > 2σ(*I*)
*R*_int_ = 0.040

### Refinement  

*R*[*F* ^2^ > 2σ(*F* ^2^)] = 0.045
*wR*(*F* ^2^) = 0.131
*S* = 1.03
2371 reflections
210 parameters
H-atom parameters constrained
Δρ_max_ = 0.25 e Å^−3^
Δρ_min_ = −0.21 e Å^−3^
Absolute structure: 957 Friedel pairs; Flack (1983 ▸)
Absolute structure parameter: 0.1 (2)

### 

Data collection: *APEX2* (Bruker, 2013 ▸); cell refinement: *SAINT* (Bruker, 2013 ▸); data reduction: *SAINT*; program(s) used to solve structure: *SHELXS97* (Sheldrick, 2008 ▸); program(s) used to refine structure: *SHELXL97* (Sheldrick, 2008 ▸); molecular graphics: *Mercury* (Macrae *et al.*, 2008 ▸); software used to prepare material for publication: *SHELXL97* and *PLATON* (Spek, 2009 ▸).

## Supplementary Material

Crystal structure: contains datablock(s) global, I. DOI: 10.1107/S2056989015019325/su5224sup1.cif

Structure factors: contains datablock(s) I. DOI: 10.1107/S2056989015019325/su5224Isup2.hkl

Supporting information file. DOI: 10.1107/S2056989015019325/su5224Isup3.cml

**Fig. 1.**
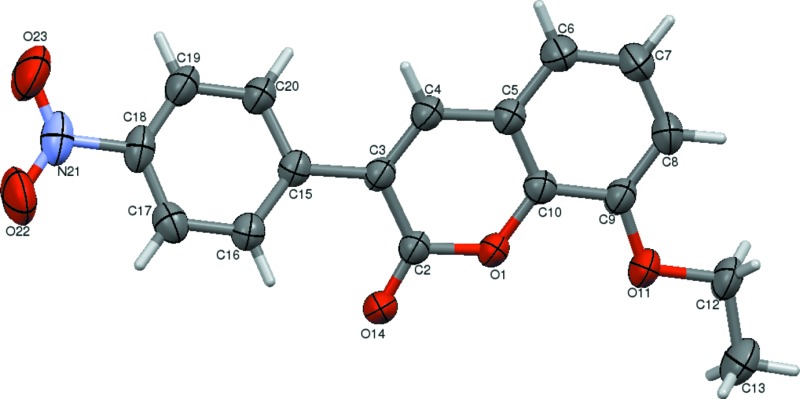
A view of the molecular structure of the title compound, with atom labelling. Displacement ellipsoids are drawn at the 50% probability level.

**Fig. 2.**
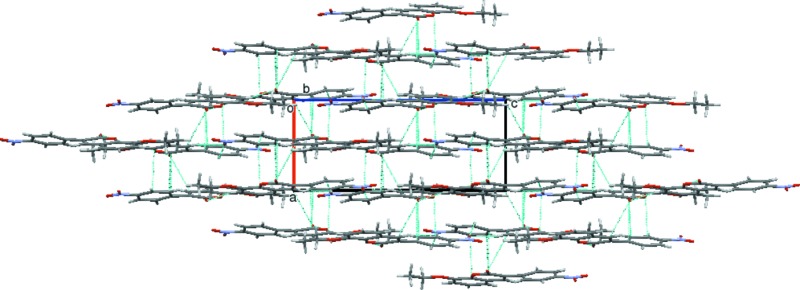
A viewed along the b axis of the crystal packing of the title compound.

. DOI: 10.1107/S2056989015019325/su5224fig1.tif
A view of the mol­ecular structure of the title compound, with atom labelling. Displacement ellipsoids are drawn at the 50% probability level.

b . DOI: 10.1107/S2056989015019325/su5224fig2.tif
A viewed along the *b* axis of the crystal packing of the title compound.

CCDC reference: 1430858

Additional supporting information:  crystallographic information; 3D view; checkCIF report

## Figures and Tables

**Table 1:** Hydrogen-bond geometry (, )

*D*H*A*	*D*H	H*A*	*D* *A*	*D*H*A*
C4H4O22^i^	0.93	2.53	3.453(2)	171
C8H8O14^ii^	0.93	2.31	3.226(2)	166
C20H20O22^i^	0.93	2.52	3.275(3)	138

Symmetry codes: (i) 
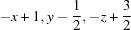
; (ii) 
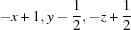
.

## supplementary crystallographic information

### S1. Comment

Coumarin derivatives are found to be one of the major groups of compounds used as fluorescent brighteners (Tian *et al.*, 2000) or fluorescent dyes and they have exhibited good bleed fastness and durability for coating plastics or in acrylic lacquers. Several natural or synthetic coumarins with various hydroxyl and other substituents were found to inhibit lipid peroxidation and to scavenge hydroxyl radicals and superoxide anions (Naveen *et al.*, 2007). As a part of our ongoing research on coumarins (Naveen *et al.*, 2006a,b), we report herein on the synthesis, characterization and crystal structure of the title compound. The compound is being assessed for biological activity.

The molecular structure of the title compound is shown in Fig. 1. The coumarin ring is essentially planar with the two axially fused rings forming a dihedral angle of 0.45 (10) °, while the 4-nitro­phenyl ring makes a dihedral angle of 25.27 (9) Å with the coumarin mean plane. The nitro group is almost planar to the phenyl ring to which it is attached with a dihedral angle of 4.3 (3) °. The eth­oxy group is inclined to the coumarin ring plane by 4.1 (2) °.

Electron delocalization was found at the short C3—C4 bond with a bond length of 1.354 (2) Å. Here as well as in other coumarin compounds reported earlier an important asymmetry in the O—C—O bond angle was detected [O1—C2—O14 = 116.10 (14)° and O14—C2—C3 = 126.14 (15)°]. The bond angles, O1—C10—C9 and C4—C5—C6, at the junction of the two rings in the coumarin moiety are 117.97 (15)° and 123.44 (16)° respectively.

In the crystal, molecules are linked via C–H···O hydrogen bonds forming sheets in the *bc* plane. The sheets are linked via π-π inter­actions [Cg1···Cg3^i^ = 3.5688 (13) Å; Cg1···Cg3^ii^ = 3.75114 (13) Å; Cg1 and Cg3 are the centroids of rings O1/C2—C5/C10 and C15—C20; symmetry codes: (i) x-1/2, -y+3/2, -z+1; (ii) x+1/2, -y+3/2, -z+1] forming a three-dimensional structure (Table 1 and Fig. 2)

### S2. Synthesis and crystallization

A mixture of 0.512 g m (3.083 mmol) of 3-eth­oxy­salicyl­aldehyde and 0.50 g m (3.083 mmol) of 4-nitro phenyl­aceto­nitrile were dissolved in ethanol (25 ml), followed by the addition of 0.525 g m (6.16 mmol) of piperidine and then the reaction mixture was stirred at room temperature for 3 h. The completion of the reaction was monitored by thin layer chromatography [petroleum ether and ethyl acetate (8:2 *v*/*v*)]. After completion the reaction mixture was filtered and washed with di­ethyl­ether giving a yellow precipitation. This product was refluxed with 10% acetic acid for 2 s and then the crude product was filtered and washed with water. It was further purified by recrystallization using acetone as solvent to give yellow crystals of the title compound in good yield (m.p.: 475-477 K; yield: 91%). ^1^H NMR(400 MHz, DMSO-d_6_): δ = 8.47 (s, 1H, Ar—H), 8.34 (dd, J= 2.08 Hz, 6.94 Hz, 2H, Ar—H), 8.05 (dd, J=2.0 Hz, 6.96 Hz, 2H, Ar—H), 7.34–7.36(m, 3H, Ar—H), 4.22(q, J= 6.92 Hz, 2H, CH2), 1.43(t, J=6.96 Hz,3*H*, CH_3_). ^13^C NMR (400 MHz, DMSO-d_6_): δ = 45.554, 142.927, 145.554, 142.927, 129.804, 124.807, 123.375, 120.205, 119.868, 115.603, 64.470, 14.63. IR (KBr) (v_max_/cm^-1^): 2925 (C—H), 1700 (C=O), 1346 (N—O), 1097 (C—O—C). Mass spectra gave a molecular ion peak at m/*z* = 311.4[*M*^+^].

### S3. Refinement

Crystal data, data collection and structure refinement details are summarized in Table 2. The hydrogen atom were fixed geometrically (C—H= 0.93–0.96 Å) and allowed to ride on their parent atoms with *U*_iso_(H) =1.5*U*_eq_(*C*-methyl) and = 1.2*U*_eq_(C) for other H atoms.

### Crystal data

**Table:**

C_17_H_13_NO_5_	*F*(000) = 648
*M**_r_* = 311.28	*D*_x_ = 1.395 Mg m^−^^3^
Orthorhombic, *P*2_1_2_1_2_1_	Cu *K*α radiation, λ = 1.54178 Å
Hall symbol: P 2ac 2ab	Cell parameters from 2371 reflections
*a* = 6.8118 (9) Å	θ = 5.6–64.1°
*b* = 13.6726 (18) Å	µ = 0.87 mm^−^^1^
*c* = 15.909 (2) Å	*T* = 296 K
*V* = 1481.7 (3) Å^3^	Prism, yellow
*Z* = 4	0.29 × 0.26 × 0.21 mm

### Data collection

**Table:**

Bruker X8 Proteum diffractometer	2371 independent reflections
Radiation source: Bruker MicroStar microfocus rotating anode	2225 reflections with *I* > 2σ(*I*)
Helios multilayer optics monochromator	*R*_int_ = 0.040
Detector resolution: 18.4 pixels mm^-1^	θ_max_ = 64.1°, θ_min_ = 5.6°
φ and ω scans	*h* = −7→6
Absorption correction: multi-scan (*SADABS*; Bruker, 2013)	*k* = −15→15
*T*_min_ = 0.786, *T*_max_ = 0.838	*l* = −18→18
6729 measured reflections	

### Refinement

**Table:**

Refinement on *F*^2^	Hydrogen site location: inferred from neighbouring sites
Least-squares matrix: full	H-atom parameters constrained
*R*[*F*^2^ > 2σ(*F*^2^)] = 0.045	*w* = 1/[σ^2^(*F*_o_^2^) + (0.103*P*)^2^] where *P* = (*F*_o_^2^ + 2*F*_c_^2^)/3
*wR*(*F*^2^) = 0.131	(Δ/σ)_max_ = 0.003
*S* = 1.03	Δρ_max_ = 0.25 e Å^−^^3^
2371 reflections	Δρ_min_ = −0.21 e Å^−^^3^
210 parameters	Extinction correction: *SHELXL*, FC^*^=KFC[1+0.001XFC^2^Λ^3^/SIN(2Θ)]^-1/4^
0 restraints	Extinction coefficient: 0.0100 (19)
Primary atom site location: structure-invariant direct methods	Absolute structure: 957 Friedel pairs; Flack (1983)
Secondary atom site location: difference Fourier map	Absolute structure parameter: 0.1 (2)

### Special details

**Table:**

Experimental. Commercially available chemicals were used directly as received. ^1^H NMR was recorded at 400 MHz in Dimethylsulfoxide (DMSO-d_6_). ^13^C NMR was recorded at 400 MHz in DMSO-d_6_. Mass spectra was recorded on a Jeol SX 102=DA-6000 (10 kV) fast atom bombardment (FAB) mass spectrometer and IR spectra was recorded on a Nicolet 5700 F T—IR instrument as KBr discs.
Geometry. Bond distances, angles *etc*. have been calculated using the rounded fractional coordinates. All su's are estimated from the variances of the (full) variance-covariance matrix. The cell e.s.d.'s are taken into account in the estimation of distances, angles and torsion angles
Refinement. Refinement on *F*^2^ for ALL reflections except those flagged by the user for potential systematic errors. Weighted *R*-factors *wR* and all goodnesses of fit *S* are based on *F*^2^, conventional *R*-factors *R* are based on *F*, with *F* set to zero for negative *F*^2^. The observed criterion of *F*^2^ > σ(*F*^2^) is used only for calculating -*R*-factor-obs *etc*. and is not relevant to the choice of reflections for refinement. *R*-factors based on *F*^2^ are statistically about twice as large as those based on *F*, and *R*-factors based on ALL data will be even larger.

### Fractional atomic coordinates and isotropic or equivalent isotropic displacement parameters (Å^2^)

**Table:**

	*x*	*y*	*z*	*U*_iso_*/*U*_eq_	
O1	0.5699 (2)	0.71184 (8)	0.34374 (7)	0.0409 (4)	
O11	0.5487 (2)	0.63066 (10)	0.19274 (7)	0.0541 (5)	
O14	0.6120 (2)	0.84884 (8)	0.41311 (7)	0.0515 (5)	
O22	0.5085 (3)	0.97085 (14)	0.82793 (11)	0.0869 (7)	
O23	0.5615 (4)	0.83031 (16)	0.88337 (9)	0.0860 (7)	
N21	0.5394 (3)	0.88318 (16)	0.82255 (11)	0.0613 (7)	
C2	0.5811 (3)	0.76190 (12)	0.41836 (10)	0.0384 (5)	
C3	0.5552 (3)	0.70612 (11)	0.49641 (10)	0.0371 (5)	
C4	0.5162 (3)	0.60920 (12)	0.49082 (10)	0.0404 (5)	
C5	0.5066 (3)	0.55829 (13)	0.41241 (11)	0.0418 (5)	
C6	0.4682 (4)	0.45725 (14)	0.40574 (12)	0.0552 (7)	
C7	0.4586 (4)	0.41549 (14)	0.32849 (12)	0.0600 (7)	
C8	0.4848 (4)	0.47095 (14)	0.25537 (12)	0.0555 (7)	
C9	0.5232 (3)	0.56989 (14)	0.25975 (11)	0.0440 (6)	
C10	0.5335 (3)	0.61330 (13)	0.33952 (11)	0.0385 (5)	
C12	0.5377 (4)	0.58696 (15)	0.10978 (10)	0.0560 (7)	
C13	0.5582 (5)	0.66749 (19)	0.04725 (12)	0.0751 (9)	
C15	0.5625 (3)	0.75681 (13)	0.57930 (11)	0.0378 (5)	
C16	0.5182 (3)	0.85604 (13)	0.59012 (11)	0.0431 (5)	
C17	0.5132 (3)	0.89769 (14)	0.66921 (12)	0.0489 (6)	
C18	0.5499 (3)	0.83947 (15)	0.73796 (11)	0.0484 (6)	
C19	0.5985 (4)	0.74317 (15)	0.73029 (11)	0.0528 (7)	
C20	0.6066 (3)	0.70218 (14)	0.65155 (10)	0.0486 (6)	
H4	0.49480	0.57420	0.54010	0.0480*	
H6	0.44980	0.41960	0.45380	0.0660*	
H7	0.43410	0.34880	0.32400	0.0720*	
H8	0.47620	0.44070	0.20310	0.0670*	
H12A	0.41270	0.55400	0.10250	0.0670*	
H12B	0.64210	0.53950	0.10250	0.0670*	
H13A	0.45240	0.71320	0.05420	0.1120*	
H13B	0.55450	0.64080	−0.00850	0.1120*	
H13C	0.68110	0.70040	0.05580	0.1120*	
H16	0.49170	0.89450	0.54330	0.0520*	
H17	0.48550	0.96380	0.67580	0.0590*	
H19	0.62580	0.70580	0.77770	0.0630*	
H20	0.64200	0.63680	0.64600	0.0580*	

### Atomic displacement parameters (Å^2^)

**Table:**

	*U*^11^	*U*^22^	*U*^33^	*U*^12^	*U*^13^	*U*^23^
O1	0.0562 (8)	0.0396 (7)	0.0269 (6)	0.0029 (6)	−0.0021 (6)	0.0005 (5)
O11	0.0836 (11)	0.0500 (8)	0.0286 (6)	0.0015 (7)	−0.0015 (6)	−0.0034 (5)
O14	0.0842 (11)	0.0360 (6)	0.0344 (6)	−0.0016 (6)	−0.0087 (7)	0.0027 (5)
O22	0.1200 (16)	0.0747 (11)	0.0659 (10)	−0.0020 (11)	0.0099 (11)	−0.0370 (9)
O23	0.1136 (16)	0.1095 (13)	0.0350 (8)	0.0045 (13)	−0.0005 (9)	−0.0103 (9)
N21	0.0599 (11)	0.0818 (14)	0.0423 (10)	−0.0075 (10)	0.0042 (9)	−0.0213 (9)
C2	0.0450 (10)	0.0401 (9)	0.0301 (8)	0.0039 (7)	−0.0032 (8)	−0.0012 (7)
C3	0.0418 (10)	0.0411 (9)	0.0285 (8)	0.0045 (8)	−0.0017 (8)	−0.0005 (7)
C4	0.0491 (10)	0.0430 (9)	0.0291 (8)	0.0027 (9)	0.0004 (7)	0.0012 (7)
C5	0.0506 (10)	0.0424 (9)	0.0325 (8)	0.0024 (8)	−0.0013 (8)	−0.0007 (7)
C6	0.0810 (15)	0.0430 (9)	0.0417 (10)	−0.0023 (10)	0.0016 (11)	0.0015 (8)
C7	0.0931 (17)	0.0382 (10)	0.0487 (10)	−0.0053 (10)	−0.0022 (12)	−0.0052 (8)
C8	0.0799 (15)	0.0470 (10)	0.0397 (9)	0.0034 (10)	−0.0059 (10)	−0.0128 (8)
C9	0.0552 (12)	0.0485 (10)	0.0282 (8)	0.0061 (9)	−0.0020 (8)	−0.0017 (7)
C10	0.0433 (9)	0.0383 (9)	0.0339 (8)	0.0044 (8)	−0.0014 (8)	−0.0014 (7)
C12	0.0765 (15)	0.0636 (12)	0.0279 (9)	0.0073 (11)	−0.0024 (9)	−0.0081 (8)
C13	0.107 (2)	0.0833 (15)	0.0351 (11)	−0.0015 (16)	0.0019 (13)	−0.0004 (10)
C15	0.0382 (9)	0.0433 (9)	0.0318 (8)	0.0011 (7)	−0.0010 (7)	−0.0026 (7)
C16	0.0500 (10)	0.0432 (9)	0.0360 (9)	−0.0011 (8)	−0.0014 (8)	−0.0033 (7)
C17	0.0528 (11)	0.0435 (10)	0.0504 (11)	−0.0023 (9)	0.0037 (10)	−0.0108 (8)
C18	0.0473 (11)	0.0645 (12)	0.0334 (9)	−0.0062 (10)	0.0010 (8)	−0.0138 (8)
C19	0.0649 (13)	0.0621 (12)	0.0314 (9)	0.0026 (10)	−0.0051 (9)	−0.0008 (8)
C20	0.0651 (13)	0.0475 (10)	0.0333 (9)	0.0055 (10)	−0.0048 (9)	−0.0014 (8)

### Geometric parameters (Å, º)

**Table:**

O1—C2	1.372 (2)	C15—C16	1.401 (3)
O1—C10	1.372 (2)	C15—C20	1.403 (2)
O11—C9	1.363 (2)	C16—C17	1.382 (3)
O11—C12	1.451 (2)	C17—C18	1.376 (3)
O14—C2	1.210 (2)	C18—C19	1.363 (3)
O22—N21	1.220 (3)	C19—C20	1.373 (2)
O23—N21	1.217 (3)	C4—H4	0.9300
N21—C18	1.474 (3)	C6—H6	0.9300
C2—C3	1.468 (2)	C7—H7	0.9300
C3—C4	1.354 (2)	C8—H8	0.9300
C3—C15	1.491 (2)	C12—H12A	0.9700
C4—C5	1.430 (2)	C12—H12B	0.9700
C5—C6	1.410 (3)	C13—H13A	0.9600
C5—C10	1.394 (3)	C13—H13B	0.9600
C6—C7	1.357 (3)	C13—H13C	0.9600
C7—C8	1.400 (3)	C16—H16	0.9300
C8—C9	1.380 (3)	C17—H17	0.9300
C9—C10	1.403 (3)	C19—H19	0.9300
C12—C13	1.490 (3)	C20—H20	0.9300
			
C2—O1—C10	122.84 (13)	N21—C18—C19	119.02 (17)
C9—O11—C12	117.00 (15)	C17—C18—C19	122.13 (17)
O22—N21—O23	123.3 (2)	C18—C19—C20	119.05 (17)
O22—N21—C18	118.08 (18)	C15—C20—C19	121.42 (18)
O23—N21—C18	118.6 (2)	C3—C4—H4	119.00
O1—C2—O14	116.10 (14)	C5—C4—H4	119.00
O1—C2—C3	117.77 (14)	C5—C6—H6	120.00
O14—C2—C3	126.14 (15)	C7—C6—H6	120.00
C2—C3—C4	118.45 (14)	C6—C7—H7	119.00
C2—C3—C15	120.19 (14)	C8—C7—H7	119.00
C4—C3—C15	121.30 (15)	C7—C8—H8	120.00
C3—C4—C5	122.85 (15)	C9—C8—H8	120.00
C4—C5—C6	123.44 (16)	O11—C12—H12A	110.00
C4—C5—C10	117.19 (16)	O11—C12—H12B	110.00
C6—C5—C10	119.36 (16)	C13—C12—H12A	110.00
C5—C6—C7	119.30 (18)	C13—C12—H12B	110.00
C6—C7—C8	121.24 (18)	H12A—C12—H12B	109.00
C7—C8—C9	120.88 (17)	C12—C13—H13A	109.00
O11—C9—C8	125.63 (16)	C12—C13—H13B	109.00
O11—C9—C10	116.32 (16)	C12—C13—H13C	109.00
C8—C9—C10	118.04 (17)	H13A—C13—H13B	110.00
O1—C10—C5	120.86 (15)	H13A—C13—H13C	109.00
O1—C10—C9	117.97 (15)	H13B—C13—H13C	109.00
C5—C10—C9	121.17 (17)	C15—C16—H16	119.00
O11—C12—C13	107.35 (16)	C17—C16—H16	119.00
C3—C15—C16	123.50 (16)	C16—C17—H17	121.00
C3—C15—C20	118.97 (15)	C18—C17—H17	121.00
C16—C15—C20	117.46 (16)	C18—C19—H19	120.00
C15—C16—C17	121.10 (17)	C20—C19—H19	121.00
C16—C17—C18	118.76 (18)	C15—C20—H20	119.00
N21—C18—C17	118.84 (18)	C19—C20—H20	119.00
			
C10—O1—C2—O14	−179.25 (17)	C6—C5—C10—C9	−0.1 (3)
C10—O1—C2—C3	0.8 (3)	C4—C5—C10—C9	−178.89 (19)
C2—O1—C10—C5	−0.3 (3)	C4—C5—C6—C7	178.9 (2)
C2—O1—C10—C9	179.35 (18)	C10—C5—C6—C7	0.1 (4)
C12—O11—C9—C8	−1.1 (3)	C4—C5—C10—O1	0.8 (3)
C12—O11—C9—C10	−179.77 (19)	C5—C6—C7—C8	−0.4 (4)
C9—O11—C12—C13	176.8 (2)	C6—C7—C8—C9	0.6 (4)
O23—N21—C18—C17	176.1 (2)	C7—C8—C9—C10	−0.5 (4)
O22—N21—C18—C19	175.2 (2)	C7—C8—C9—O11	−179.2 (2)
O22—N21—C18—C17	−3.8 (3)	O11—C9—C10—C5	179.02 (18)
O23—N21—C18—C19	−4.9 (3)	C8—C9—C10—O1	−179.4 (2)
O1—C2—C3—C4	−1.9 (3)	C8—C9—C10—C5	0.3 (3)
O1—C2—C3—C15	−178.98 (17)	O11—C9—C10—O1	−0.7 (3)
O14—C2—C3—C15	1.1 (3)	C3—C15—C16—C17	175.17 (19)
O14—C2—C3—C4	178.2 (2)	C20—C15—C16—C17	−1.7 (3)
C2—C3—C15—C20	−157.80 (19)	C3—C15—C20—C19	−174.2 (2)
C2—C3—C15—C16	25.4 (3)	C16—C15—C20—C19	2.8 (3)
C2—C3—C4—C5	2.5 (3)	C15—C16—C17—C18	−0.9 (3)
C4—C3—C15—C16	−151.6 (2)	C16—C17—C18—N21	−178.37 (19)
C4—C3—C15—C20	25.2 (3)	C16—C17—C18—C19	2.6 (3)
C15—C3—C4—C5	179.53 (19)	N21—C18—C19—C20	179.4 (2)
C3—C4—C5—C6	179.3 (2)	C17—C18—C19—C20	−1.6 (4)
C3—C4—C5—C10	−1.9 (3)	C18—C19—C20—C15	−1.2 (4)
C6—C5—C10—O1	179.6 (2)		

### Hydrogen-bond geometry (Å, º)

**Table:**

*D*—H···*A*	*D*—H	H···*A*	*D*···*A*	*D*—H···*A*
C4—H4···O22^i^	0.93	2.53	3.453 (2)	171
C8—H8···O14^ii^	0.93	2.31	3.226 (2)	166
C20—H20···O22^i^	0.93	2.52	3.275 (3)	138

Symmetry codes: (i) −*x*+1, *y*−1/2, −*z*+3/2; (ii) −*x*+1, *y*−1/2, −*z*+1/2.
