# Supplementary figures and images for: Crystal structure of 8-eth­oxy-3-(4-nitro­phen­yl)-2H-chromen-2-one
Source: Acta Crystallogr E Crystallogr Commun. 2015 Oct 17;71(Pt 11):o860–1. doi: 10.1107/S2056989015019325 (PMC4645040; doi:10.1107/S2056989015019325)

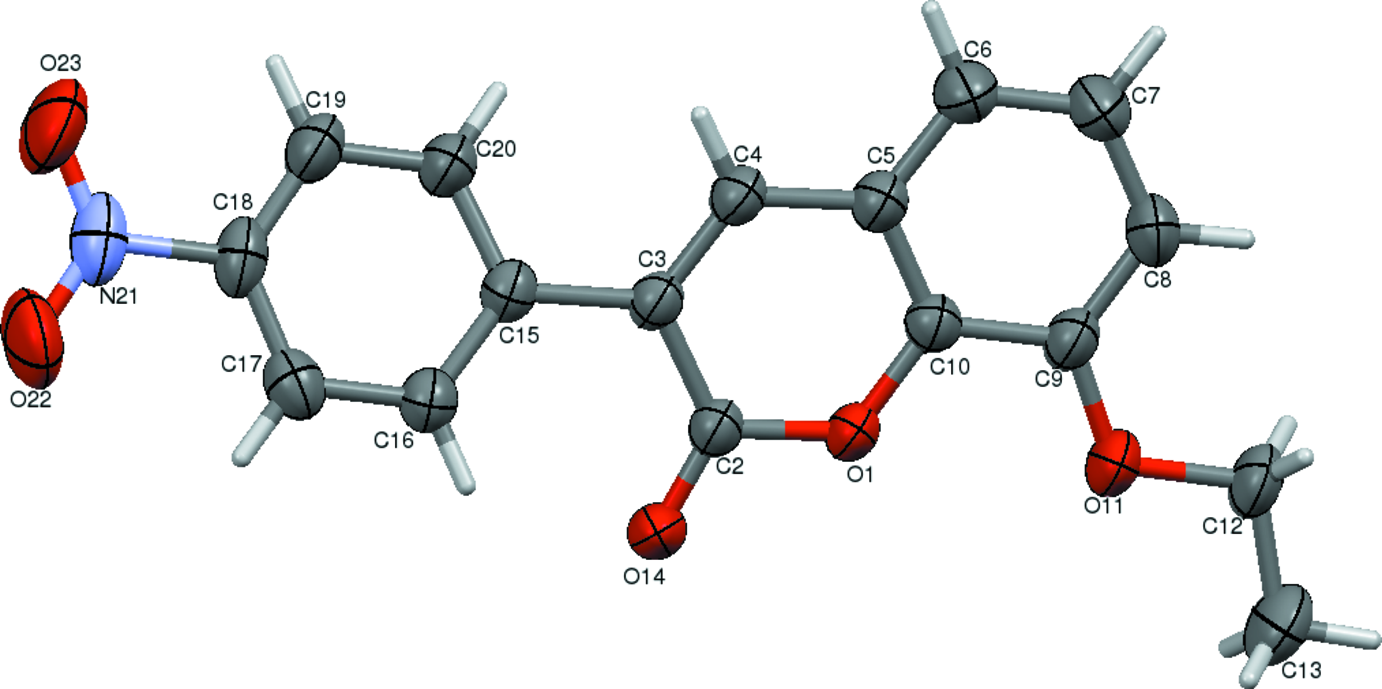

Supplement: Supplementary file 4 [file e-71-0o860-fig1.tif]

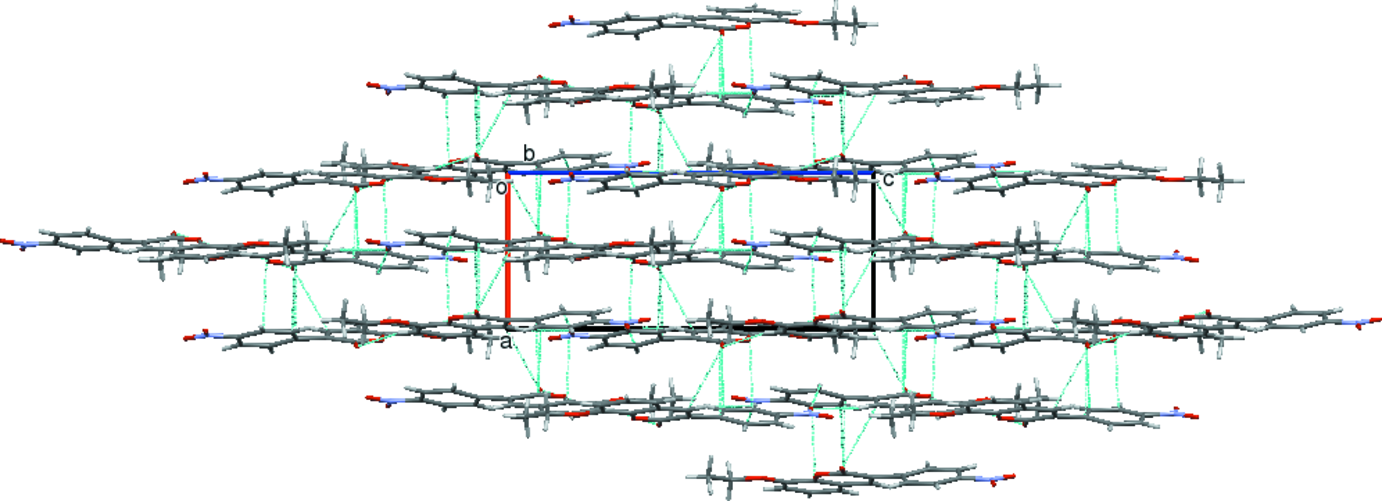

Supplement: Supplementary file 5 [file e-71-0o860-fig2.tif]
